# Genome and transcriptome analysis of *Bacillus velezensis* BS‐37, an efficient surfactin producer from glycerol, in response to d‐/l‐leucine

**DOI:** 10.1002/mbo3.794

**Published:** 2019-02-22

**Authors:** Dayuan Zhou, Fangxiang Hu, Junzhang Lin, Weidong Wang, Shuang Li

**Affiliations:** ^1^ College of Biotechnology and Pharmaceutical Engineering Nanjing Tech University Nanjing China; ^2^ Oil Production Research Institute Shengli Oil Field Ltd. Co. SinoPEC Dongying China; ^3^ Jiangsu National Synergetic Innovation Center for Advanced Materials (SICAM) Nanjing Tech University Nanjing China

**Keywords:** biofilm, genome, leucine, surfactin, transcriptome

## Abstract

Surfactin is one of the most widely studied biosurfactants due to its many potential applications in different fields. In the present study, *Bacillus velezensis* BS‐37, initially identified as a strain of *Bacillus subtilis*, was used to efficiently produce surfactin with the addition of glycerol, an inexpensive by‐product of biodiesel production. After 36 hr of growth in glycerol medium, the total surfactin concentration reached more than 1,000 mg/L, which was two times higher than that in sucrose medium. Moreover, the addition of l‐ and d‐Leu to the culture medium had opposite effects on surfactin production by BS‐37. While surfactin production increased significantly to nearly 2,000 mg/L with the addition of 10 mM l‐Leu, it was dramatically reduced to about 250 mg/L with the addition of 10 mM d‐Leu. To systemically elucidate the mechanisms influencing the efficiency of this biosynthesis process, we sequenced the genome of BS‐37 and analyzed changes of the transcriptome in glycerol medium in response to d‐/l‐leucine. The RPKM analysis of the transcriptome of BS‐37 showed that the transcription levels of genes encoding modular surfactin synthase, the glycerol utilization pathway, and branched‐chain amino acid (BCAA) synthesis pathways were all at a relatively high level, which may offered an explanation why this strain can efficiently use glycerol to produce surfactin with a high yield. Neither l‐Leu nor d‐Leu had a significant effect on the expression of genes in these pathways, indicating that l‐Leu plays an important role as a precursor or substrate involved in surfactin production, while d‐Leu appears to act as a competitive inhibitor. The results of the present study provide new insights into the synthesis of surfactin and ways of its regulation, and enrich the genomic and transcriptomic resources available for the construction of high‐producing strains.

## 1 INTRODUCTION

Biosurfactants, for their excellent surface activity, temperature tolerance, and biodegradability, have received great attention in the past decade. However, apart from a few biosurfactants such as sophorolipids, rhamnolipids, and mannosyl erythritol lipids (Chen, Juang, & Wei, 2015; Desai & Banat, 1997), their industrial application is largely restricted. Surfactin, a lipopeptide biosurfactant produced by *Bacillus* strains, is one of the most surface‐active biosurfactants, and it has potential applications in medicine, agriculture, and microbial enhanced oil recovery (MEOR) (Ashish & Debnath, 2018; Paraszkiewicz et al., 2017; Rodrigues, Banat, Teixeira, & Oliveira, 2006). However, due to its high production cost, its industrial applications are still underdeveloped. Therefore, improving its yield and reducing its production cost will be of great significance. Most of the efficient surfactin producers reported to date prefer sucrose as the carbon source (Jiao et al., 2017; Yan, Wu, & Xu, 2017). By contrast, glycerol, which is a by‐product of biodiesel production, is a widely available and inexpensive source of carbon, making its conversion into value‐added products of great interest (Clomburg & Gonzalez, 2013; Faria et al., 2011). Thus, producing surfactin efficiently utilizing glycerol as carbon source can not only reduce the production cost, but also solve the problem of glycerol valorization in the production of biodiesel.

**Figure 1:**
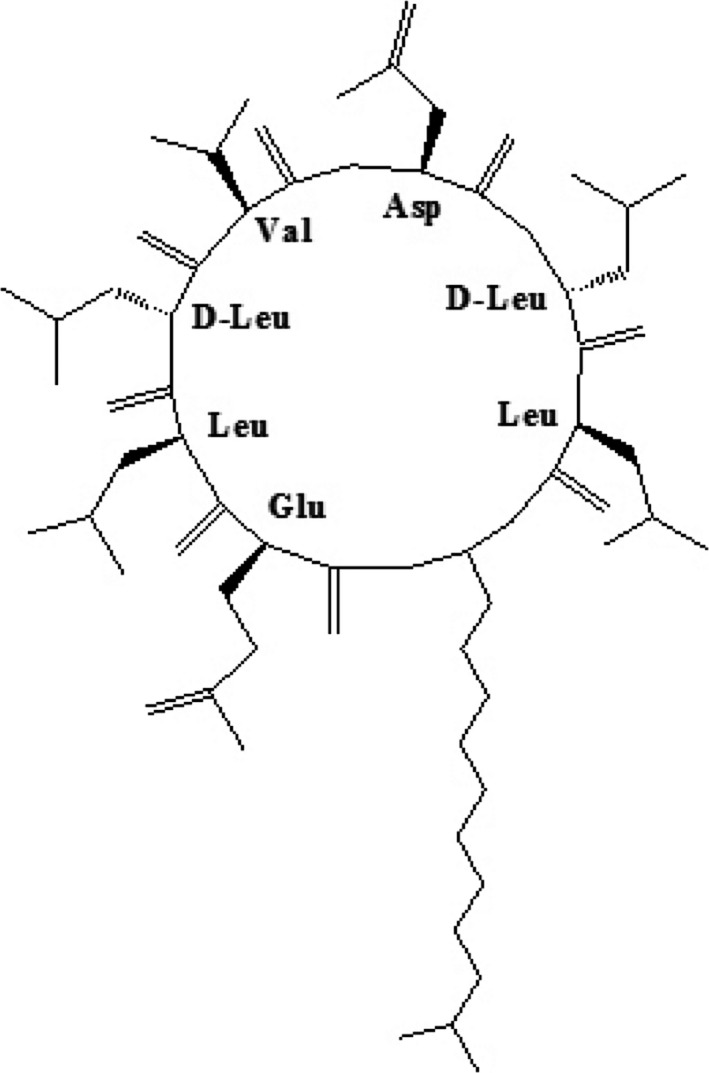
Schematic representation of surfactin (Coutte et al., 2015)

Surfactin is synthesized via a complex mechanism catalyzed by a nonribosomal peptide synthetase (NRPS), which is encoded by the srfA operon (srfAA, srfAB, srfAC, srfAD) (Nakano et al., 1991). These enzymes preferentially use amino acid and fatty acid residues present in the cytoplasm of the cell as substrates. The hydrophobic tail of surfactin is a β‐hydroxylated fatty acid chain with different lengths and isoforms, and the hydrophilic head is a circular heptapeptide containing l‐glutamic (l‐Glu), l‐aspartic (l‐Asp), l‐valine (l‐Val), l‐leucine (l‐Leu), and d‐leucine (d‐Leu) (Seydlova & Svobodova, 2008; Yang, Li, Li, Yu, & Shen, 2015). Therefore, amino acids and fatty acids are of great importance for the synthesis of surfactin, regardless of the synthetic mechanism or structural characteristics of the surfactin subtype. Liu, Yang, Yang, Ye, & Mu, 2012 reported that the addition of different amino acids to the cultures had a significant influence on the proportion of surfactin variants with different fatty acids. The peptide moiety contains two l‐Leu and two d‐Leu residues (Figure 1), and the content of Leu in cells is crucial for the synthesis of surfactin (Etchegaray et al., 2017). Moreover, the research of Coutte et al. indicated that leucine overproduction significantly improved the production of surfactin in recombinant *Bacillus subtilis* 168 derivatives (Coutte et al., 2015).

In this study, *Bacillus velezensis* BS‐37 was found to efficiently produce surfactin at a very high level with the addition of glycerol. Interestingly, the addition of l‐ and d‐Leu to the culture medium as nitrogen source had opposite effects on surfactin production by BS‐37. To better understand the strain BS‐37 and the effects of d‐/l‐Leu on surfactin synthesis, genome sequencing in combination with global transcriptome analysis was employed. The transcription levels of genes related to surfactin biosynthesis, including the modular surfactin synthetase, glycerol utilization pathway, branched‐chain amino acid (BCAA) synthesis pathways, and the branched fatty acid (FA) metabolic pathway, were further studied. These results will hopefully provide a theoretical basis for later genetic manipulation of *B. velezensis* BS‐37.

## 2 MATERIALS AND METHODS

### 2.1 Bacterial strains and growth conditions

*Bacillus velezensis* BS‐37 is a mutant derivative of strain 723, which was isolated from petroleum‐contaminated soil from the Shengli Oil Field, China (Zhu, Xu, Jiang, Huang, & Li, 2014). It was cultivated in 250‐ml flasks containing 50 ml of minimal medium (0.2% NH_4_NO_3_, 1% KH_2_PO_4_•3H_2_O, 0.02% MgSO_4_•7H_2_O, 0.002% FeSO_4_•7H_2_O) supplied with 2% glycerol (MMG) or 2% sucrose (MMS). Different concentrations (0–20 mM) of d‐Leu or l‐Leu were added into MMG medium, and a group without added amino acids served as the negative control. The pH of all media was adjusted to 7.5 with 5 M HCl or 5 M NaOH. Sterilization was conducted at 121°C for 20 min. The cultures were incubated at 37°C and 200 rpm. At various time‐points, the cell density was measured by measuring the OD_600_ using a spectrometer.

### 2.2 Surfactin production assay

The surfactin production was quantified according to a published HPLC method (Zhu et al., 2014). Briefly, 1 ml samples were extracted from the shake flasks, and a cell‐free supernatant was obtained by centrifugation at 10,000 *g* for 10 min. An aliquot comprising 300 µl of the supernatant was withdrawn, diluted fivefold with methanol, and shaken for 1 min. Then, the precipitate of samples was removed through centrifugation for 2 hr at 10,000 *g* under 4°C.

Subsequently, the supernatant needed to be further filtered by a 0.22 μm nylon membrane remove impurities. The final sample was analyzed by HPLC on a U‐3000 system (Thermo Fisher Scientific, USA) equipped with a Synchronis C18 column (4.6 × 250 mm, 5 μm; Thermo Fisher Scientific) and a UV detector (Thermo Fisher Scientific). The analytes were detected at 214 nm, using 90% (*v*/*v*) methanol, 10% (*v*/*v*) water, and 0.05% trifluoroacetic acid as mobile phase with a flow rate of 0.8 ml/min. The authentic surfactin (98%) reference standard was purchased from Sigma‐Aldrich, USA. The proportions of surfactin isoforms were analyzed by comparing the peak areas observed by HPLC to the sum of areas of all surfactin peaks.

### 2.3 Genome sequencing and annotation

Before sequencing, the BS‐37 cells needed to be harvested through centrifugation for 5 min at 10,000 *g* and 4°C after cultivated for 24 hr. The isolation of genomic DNA was carried out using a Rapid Bacterial Genomic DNA Isolation Kit (GENEWIZ, China). Whole‐genome sequencing was performed using a Pacific Biosciences (PacBio, China) RSII single molecule real‐time (SMRT) sequencing technique with a 20‐kb SMARTbellTM template library. The circular chromosome was assembled by the obtained reads with the SMRT Analysis 2.3.0 software. Then, the complete genome of *B. velezensis* BS‐37 was upload to the NCBI database under the GenBank accession number CP023414. The prodigal software was used to assign coding genes from bacteria (Hyatt et al., 2010). Gene annotation of *B. velezensis*BS‐37 was performed using the National Center for Biotechnology Information (NCBI) database in conjunction with Diamond (Buchfink, Xie, & Huson, 2015). Subsequently, the functions of genes were annotated using the GO database (http://www.geneontology.org/), and the pathways were annotated using the KEGG database (http://www.genome.ad.jp/kegg/). The proteins encoded by the annotated genes were classified based on phylogeny using the COG database (http://www.ncbi.nlm.nih.gov/COG/).

### 2.4 RNA isolation, library construction, and Illumina sequencing

For RNA extraction, the cells of BS‐37 for 12 hr were harvested by centrifugation at 8,000 *g* for 5 min at 4°C, at the period of the fermentation when the production rate was the highest. The resulting pellets were immediately frozen in liquid nitrogen. Total RNA was isolated using the Trizol Reagent (Invitrogen Life Technologies, USA), according to the manufacturer's instructions. Quality and integrity were determined using a NanoDrop spectrophotometer (Thermo Scientific) and a Bioanalyzer 2100 system (Agilent, USA). To remove rRNA, the Ribo‐Zero rRNA removal kit (Illumina, San Diego, CA, USA) was employed. The synthesis of first‐strand cDNA was performed by random oligonucleotides and SuperScript III. Subsequently, polymerase I and RNase H were used to synthesize the second‐strand cDNA. The conversion of remaining overhangs to blunt ends was conducted by polymerase. The hybridization was performed after the adenylation of the 3′ ends of the DNA fragments and the combination with Illumina PE adapter oligonucleotides. To select the preferred cDNA fragments length of 300 bp, the library fragments were purified using the AMPure XP system (Beckman Coulter, Beverly, CA, USA). Illumina PCR Primer Cocktail in a 15 cycle PCR was used to selectively enrich the DNA fragments with ligated adaptor molecules on both ends. Besides, purification and quantification of products were conducted by AMPure XP system (Beckman Coulter) and Agilent high sensitivity DNA assay on a Bioanalyzer 2100 system (Agilent), respectively. Finally, the resulting library was sequenced on a NextSeq 500 platform (Illumina) by Shanghai Personal Biotechnology Co. Ltd.

### 2.5 Mapping reads to the reference genome and normalized gene expression

After removing out rRNA reads, sequencing adapters, short‐fragment reads, and other low‐quality reads, the sequencing raw reads were preprocessed. The remaining clear reads were mapped to the reference genome of BS‐37 using Bowtie 2 software based on the local alignment algorithm (Langmead & Salzberg, 2012). By calculating the RPKM value (reads per kilobase per million mapped reads), the expression levels and the gene length were normalized to the library (Frazee et al., 2015). DESeq software was used to quantify all transcripts with differential expression (Wang, Feng, Wang, Wang, & Zhang, 2010). Correcting the consequence of multiple hypothesis testing was operated by FDR (False Discovery Rate) control.

### 2.6 Reconstruction of the KEGG pathway map

The assignment of functional annotation descriptions was conducted by BLASTP (Altschul et al., 1998) in conjunction with the KEGG database (*E*‐value cutoff of 1E‐10). Subsequently, the KEGG map was manually redrawn using Adobe Illustrator CC 2014 (Adobe Systems, USA). The expressed genes which involved in the biosynthesis of surfactin were generated as heat maps with the GraphPad Prism 7 (GraphPad Software, Inc., La Jolla, CA, USA).

## 3 RESULTS AND DISCUSSION

### 3.1 Genomic analysis of the strain BS‐37

**Figure 2:**
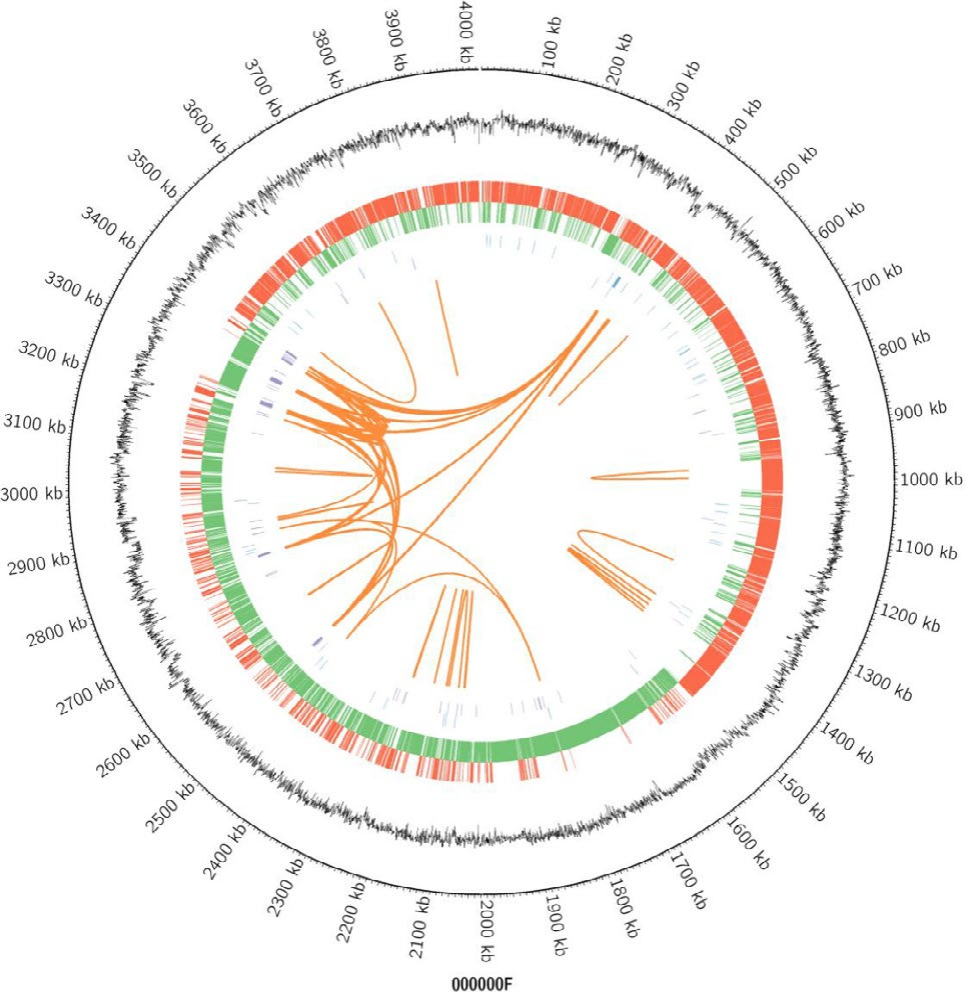
Circular representation of the *Bacillus velezensis* BS‐37 genome. The circular map consists of five circles. From the outmost circle inwards, each circle contains information about the genome regarding the G + C ratio, reverse CDS, forward CDS, rRNA/tRNA, and long segment repeats, respectively

The optimal carbon source for strain BS‐37 was found to be glycerol, and its fermentation products contained more than 50% of the desirable surfactin isoform C_15_(Liu, Lin, Wang, Huang, & Li, 2015; Yi et al., 2017). In order to investigate the genomic organization of the metabolic pathways responsible for glycerol utilization and biosynthesis of surfactin, the complete genome of strain BS‐37 was sequenced. This strain was initially incorrectly identified as a *B. subtilis*. Subsequently, it was demonstrated by complete genome sequencing that this is a strain of *B. velezensis*. The principal features of the BS‐37 genome are shown in Figure 2. The 4,013,888‐bp long BS‐37 genome is composed of a circular chromosome with an average GC content of 46.46%. The chromosome contains 3,846 CDS, 90 rRNA and 86 tRNA genes (Table 1). Among the CDS, 3,395 (88.27%) were classified into 21 “cluster of orthologous groups” (COG) functional categories. The functions of most genes were associated with important transcription and metabolism pathways, such as amino acid, carbohydrate, inorganic ion and energy production and conversion pathways (Table 2).

**Table 1:** Features of the *Bacillus velezensis* BS‐37 genome

Feature	Value
Chromosome number	1
Genome size (bp)	4,013,888
GC content (%)	46.46
Protein coding genes (CDS)	3,846
rRNA	90
tRNA	86
ncRNA	91

**Table 2:** Distribution of COG functional categories in the complete genome sequence of *Bacillus velezensis* BS‐37

COG code	Description	Gene number
F	Nucleotide transport and metabolism	81
E	Amino acid transport and metabolism	344
Q	Biosynthesis, transport and catabolism of secondary metabolites	110
I	Lipid transport and metabolism	115
B	Chromatin structure and dynamics	1
K	Transcription	281
L	Replication, recombination, and repair	132
S	Function unknown	314
G	Carbohydrate transport and metabolism	265
R	General function prediction only	446
H	Coenzyme transport and metabolism	132
J	Translation, ribosomal structure, and biogenesis	162
U	Intracellular trafficking, secretion, and vesicular transport	46
D	Cell cycle control, cell division, chromosome partitioning	35
C	Energy production and conversion	181
T	Signal transduction mechanisms	151
O	Posttranslational modification, protein turnover, chaperones	97
M	Cell wall/membrane/envelope biogenesis	192
N	Cell motility	56
P	Inorganic ion transport and metabolism	195
V	Defense mechanisms	59
Total		3,395

**Figure 3:**
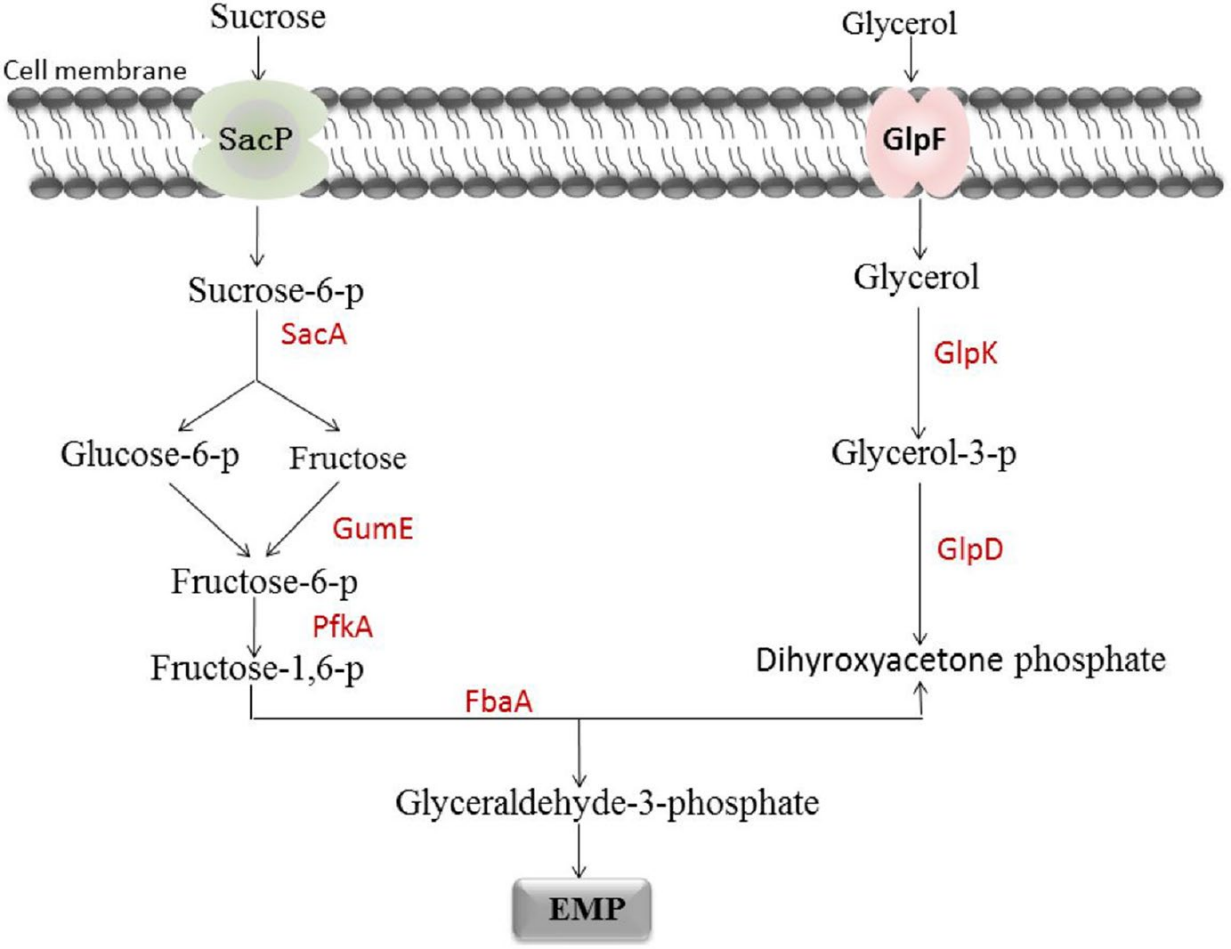
Key genes of the pathways channeling sucrose and glycerol into the glycolytic pathway of *Bacillus* sp.

The putative key genes of glycerol and sucrose metabolism in BS‐37 were identified by whole‐genome analysis (Figure 3). The amino acid sequences encoded by these genes were arranged in comparison with those of *B. subtilis* 168 and MT45. In sucrose metabolism, five key enzymes catalyzing the steps from sucrose to glyceraldehyde‐3‐phosphate were compared, revealing that sucrose transport, hydrolysis, and fructokinase are represented by different isozymes. In fact, there were large differences between strain 168 and BS‐37, with SacA, SacP, and GmuE having sequence similarities at the protein level of <80%. By contrast, the similarity with MT45 was higher than 90% in all cases. In glycerol metabolism, three key proteins were compared between 168 and MT45, and their protein sequence similarity reached over 90% (Table 3).

**Table 3:** Analysis of amino acid identity of the putative key genes for glycerol and sucrose metabolism

Function	Gene name	Gene ID	168	MT45
		(BS‐37)	Identity (%)	
Sucrose metabolism	*sacP* a	1_0012/1_3515	79/82	94/99
	*sacA* a	1_3054/1_3516	77/78	90/91
	*gmuE* a	1_2644/1_3453	67/68	92/95
	*fbaA*	1_3612	99	99
	*pfkA*	1_0543	97	99
Glycerol metabolism	*glpF*	1_2344	87	96
	*glpK*	1_2343	92	97
	*glpD*	1_2342	90	98

a Isozymes with different coding genes.

### 3.2 The biomass and surfactin production from glycerol and sucrose

**Figure 4:**
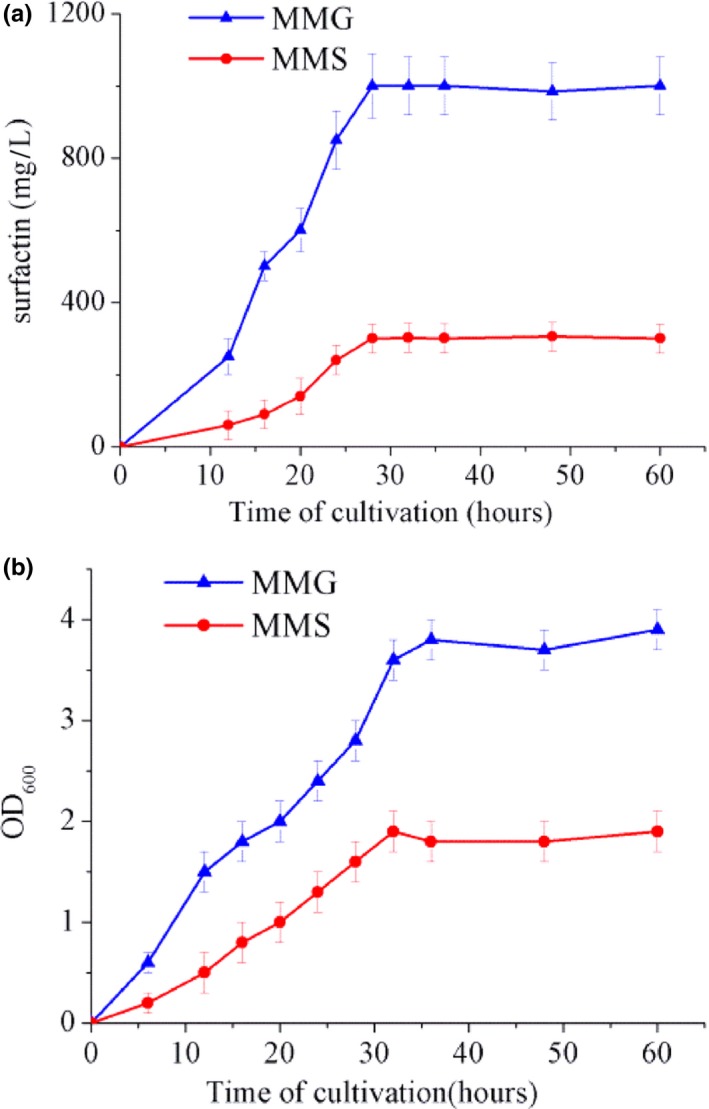
Surfactin production and cell growth in MMG and MMS media. (a) Surfactin production curve. (b) Cell growth curve

Many previously characterized bacteria, such as *B. subtilis* 168 and *Bacillus amyloliquefaciens* MT45, can utilize sucrose to produce surfactin with high efficiency, but few of these can efficiently use glycerol (Amani, Haghighi, & Keshtkar, 2013; Biniarz, Coutte, Gancel, & Lukaszewicz, 2018; Zhu, Zhang, Chen, Cai, & Lin, 2016). It is therefore notable that BS‐37 shows the opposite substrate preference. The results revealed that up to 1,000 mg/L of surfactin could be accumulated after 36 hr of fermentation in minimal medium with glycerol (MMG). By contrast, the yield only reached about 350 mg/L in minimal medium with sucrose (MMS; Figure 4a). In addition, this strain reached an OD_600_ of 3.5 in MMG, which was twice higher than in MMS (Figure 4b). These results indicate that BS‐37 prefers glycerol over sucrose in terms of both growth and efficient surfactin production. Notably, the pH was constant during the 60 hr of cultivation.

### 3.3 Effects of supplementing d‐ and l‐Leu on surfactin production

**Figure 5:**
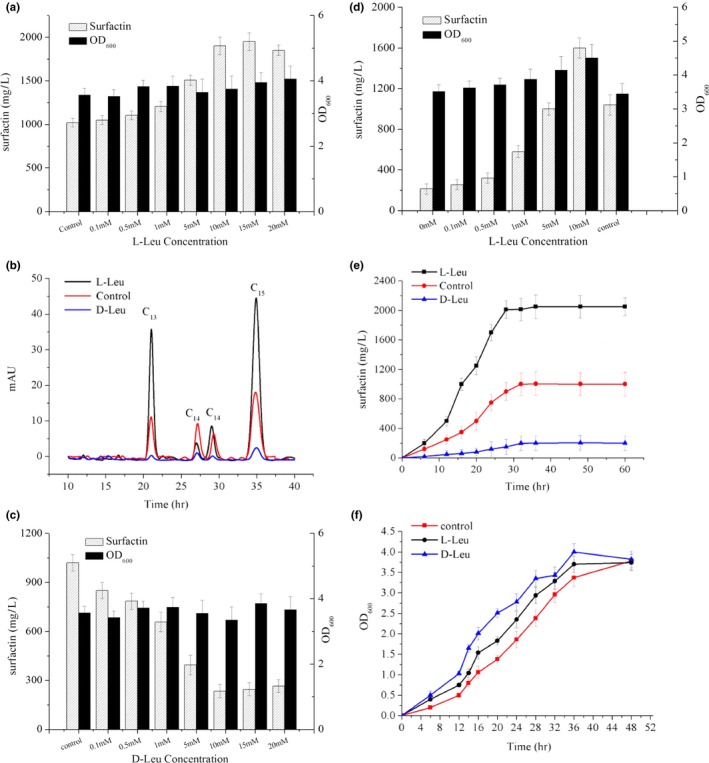
Cell growth and surfactin production of BS‐37 supplemented with d‐/l‐Leu. (a) Biomass and surfactin production in cultures with l‐Leu. (b) The proportion of surfactin variants (c) Biomass and surfactin production in cultures with d‐Leu. (d) Surfactin production in 10 mM d‐Leu medium with l‐Leu supplementation. (e) Surfactin production time course. (f) Cell growth time course

To investigate the influence of d‐/l‐Leu on the growth of BS‐37 and the yield of surfactin, a preliminary study of the addition of d‐/l‐Leu at a series of concentrations was conducted. All the production and biomass data were collected after 36 hr of fermentation. With the increase in d‐/l‐Leu concentration, surfactin production showed great differences. The surfactin yield improved with increasing concentrations of l‐Leu, and with the supplementation of 10 mM l‐Leu reached a maximum of about 2,000 mg/L, twice that of the control (Figure 5a). Adding l‐Leu increased the percentage of the surfactin fatty acid chain length variants iso‐C_13_ and iso‐C_15_ (Figure 5b), as had already been reported (Liu, Yang, Yang, Ye, & Mu, 2012). However, the addition of d‐Leu unexpectedly inhibited the production of surfactin. With the increase in d‐Leu concentration, the surfactin yield gradually decreased, reaching a low titer of 250 mg/L with the supplementation of 10 mM d‐Leu (Figure 5c). Interestingly, it was found that the inhibitory effects of 10 mM d‐Leu on surfactin accumulation can be relieved by adding 5 mM l‐Leu, and the surfactin titer was restored to 1,000 mg/L. The surfactin titer could even be increased to 1,600 mg/L by adding 10 mM l‐Leu on the basis of 10 mM d‐Leu (Figure 5d).

### 3.4 The transcriptome analysis effects of adding d‐ or l‐Leu

To reveal the transcriptomic correlates of the overproduction, we performed a transcriptome analysis based on RNA sequencing of BS‐37 under supplementation of 10 mM d‐/l‐Leu and control cultures. Samples were taken during the growth stage with the highest biomass increase and accumulation of surfactin (24 hr; Figure 5e,f). Three samples of each group were extracted and sequenced, generating over ten million reads for each sample with cleans ratios >85%. The sequence data are summarized in Table 4. Each sample had over 98% clean reads that could be matched to the reference genome, which indicated that the transcriptome data can provide sufficient support for further analysis.

**Table 4:** Summary of RNA‐seq and the reads mapped to the genome of BS‐37

Sample	Raw reads	Total mapped reads	%	Uniquely mapped reads	%	Multiple mapped reads	%
Control‐1	29,367,782	28,813,155	99.1	28,056,164	97.37	756,991	2.63
Control‐2	31,758,858	31,113,270	98.56	30,363,811	97.59	749,459	2.41
Control‐3	32,117,854	30,982,819	97.13	30,113,517	97.19	869,302	2.81
10 mM l‐Leu‐1	29,297,620	28,659,072	98.64	27,950,164	97.53	708,908	2.47
10 mM l‐Leu‐2	31,467,442	30,777,604	98.56	29,808,425	96.85	969,179	3.15
10 mM l‐Leu‐3	36,099,214	35,392,254	98.73	34,347,745	97.05	1,044,509	2.95
10 mM d‐Leu‐1	34,050,432	33,515,348	99.19	31,973,112	95.4	1,542,236	4.6
10 mM d‐Leu‐2	33,131,254	32,420,664	98.48	31,692,349	97.75	728,315	2.25
10 mM d‐Leu‐3	32,684,204	32,126,771	99.13	31,176,494	97.04	950,277	2.96

Differentially expressed genes (DEGs) in response to l‐/d‐Leu are listed in Table 5. Compared with the control, five genes were upregulated and seven genes were downregulated when adding 10 Mm l‐Leu; When 10 mM d‐Leu was added, 17 genes were upregulated and 25 were downregulated. Interestingly, the expression level of the azlBCD operon (encoding genes azlB, azlC, and azlD), which is responsible for the transport of leucine, had little difference between control and l‐/d‐Leu. Sporulation can be induced in *B. subtilis* by starvation of carbon, nitrogen, and phosphorus. In this study, four genes involved in sporulation (*cotV*, *cotX*, *rpsR*, and *cgeB*) were found to be downregulated after adding d‐Leu. Furthermore, the key genes related to amino acid and purine metabolism, such as *argD*, *argB*, *purC*, *pyrB*, *pyrR,* and *guaC*, were upregulated in the cultures with d‐Leu. These products provide the energy required for cell proliferation, offering an explanation for the higher biomass in the presence of d‐Leu compared to the control in the early stages (Table 6).

**Table 5:** Genes of *Bacillus velezensis* BS‐37 differentially expressed in response to l‐/d‐Leu

Gene	Encoding protein	Fold change ratio	*q*‐value (%)
Upregulated (expressed in response to d‐Leu)			
*argD*	Acetylornithine aminotransferase	2.26	0.04
*argB*	Acetylglutamate kinase	2.42	0.02
*argG*	Argininosuccinate synthase	2.54	0.04
*pyrC*	Dihydroorotase	2.15	0.04
*pyrB*	Aspartate carbamoyltransferase	2.26	0.04
*pyrR*	Bifunctional pyrimidine regulatory protein PyrR uracil phosphoribosyltransferase	2.06	0.02
*guaC*	GMP reductase	2.56	0.02
*rutR*	TetR family transcriptional regulator	2.08	0.04
*carA*	Carbamoyl phosphate synthase small subunit	2.57	0.03
*lepB*	Signal peptidase	2.00	0.04
*dsbB*	2‐oxoglutarate dehydrogenase	2.46	0.03
*plT*	Inorganic phosphate transporter	2.01	0.00
*mccB*	Cystathionine gamma‐synthase	2.61	0.01
*opuC*	Glycine betaine/carnitine/choline‐binding protein	2.22	0.04
*yxlA*	Hypothetical protein	3.45	0.00
*yxlD*	Putative protein	2.78	0.02
*yxwF*	Hypothetical protein	2.78	0.03
Downregulated (expressed in response to d‐Leu)			
*cgeB*	Spore maturation protein CgeB	0.04	0.04
*cotV*	Spore coat protein	0.37	0.01
*cotX*	Spore coat protein	0.42	0.02
*rpsR*	Sporulation protein YjcZ	0.25	0.00
*bioW*	6‐carboxyhexanoate‐CoA ligase	0.33	0.01
*bioA*	Adenosylmethionine‐8‐amino‐7‐oxononanoate aminotransferase	0.22	0.00
*bioF*	8‐amino‐7‐oxononanoate synthase	0.14	0.00
*bioD*	Dethiobiotin synthetase	0.14	0.00
*bioB*	Biotin synthase	0.21	0.00
*bioI*	Cytochrome P450	0.21	0.00
*liaI*	Protein iaI	0.31	0.01
*liaH*	Phage shock protein A homolog	0.33	0.01
*fadD*	Acyl‐CoA synthetase	0.45	0.04
*atoB*	Acetyl‐CoA acetyltransferase	0.43	0.03
*gerE*	LuxR family transcriptional regulator	0.34	0.01
*ywlT*	Hypothetical protein	0.37	0.03
*ywhV*	Membrane protein	0.15	0.00
*ywgA*	Hypothetical protein	0.14	0.03
*ylmF*	Uncharacterized protein	0.35	0.01
*ysrdE*	Uncharacterized protein	0.19	0.00
*ylxF*	Putative oxidoreductase	0.40	0.03
*yrzF*	Uncharacterized protein	0.16	0.00
*ywqH*	Hypothetical protein	0.02	0.02
*ysdB*	Hypothetical protein	0.01	0.01
*yitR*	Hypothetical protein	0.14	0.00
Upregulated (expressed in response to l‐Leu)			
*pgsC*	Poly‐gamma‐glutamate biosynthesis protein	3.25	0.02
*yxgK*	Hypothetical protein	2.74	0.02
*yxgC*	Hypothetical protein	2.13	0.03
*yxgB*	Hypothetical protein	2.26	0.05
*yxgM*	Hypothetical protein	2.61	0.02
Downregulated (expressed in response to l‐Leu)			
*bioA*	Adenosylmethionine‐8‐amino‐7‐oxononanoate aminotransferase	0.40	0.01
*bioF*	8‐amino‐7‐oxononanoate synthase	0.41	0.02
*bioD*	Dethiobiotin synthetase	0.34	0.01
*bioB*	Biotin synthase	0.41	0.02
*bioI*	Cytochrome P450	0.38	0.00
*clpE*	ATP‐dependent Clp protease ATP‐binding protein	0.49	0.04
*yxgH*	Uncharacterized protein	0.48	0.03

**Table 6:** Detail information on genes involved in the surfactin synthesis pathway and their expression levels

Gene	Encoding protein	RPKM	Expression intensity
*glpF*	Glycerol uptake facilitator protein	268.05	M
*glpK*	Glycerol kinase	292.79	M
*glpD*	Glycerol‐3‐phosphate dehydrogenase	3,913.68	H
*gapA*	Glyceraldehyde 3‐phosphate dehydrogenase	822.98	H
*gapA*‐*1*	Glyceraldehyde 3‐phosphate dehydrogenase	360.78	H
*pgK*	Phosphoglycerate kinase	1,263.41	H
*pgmB*	Beta‐phosphoglucomutase	19.01	L
*enO*	Enolase	829.03	H
*pyK*	Pyruvate‐kinase	741.43	H
*pdhA*	Pyruvate dehydrogenase E1 component alpha subunit	35.57	L
*PdhA*‐*1*	Pyruvate dehydrogenase E1 component alpha subunit	48.42	L
*pdhB*	Pyruvate dehydrogenase E1 component beta subunit	134.22	M
*pdhB*‐*1*	Pyruvate dehydrogenase E1 component beta subunit	42.10	L
*pdhC*	Pyruvate dehydrogenase E2 component	88.81	L
*pdhC*‐*1*	Pyruvate dehydrogenase E2 component	559.59	H
*gltA*	Citrate synthase	41.75	L
*gltA*‐*1*	Citrate synthase	1,275.28	H
*gltA*‐*2*	Citrate synthase	142.42	M
*acO*	Aconitate hydratase	596.77	H
*icD*	Isocitrate dehydrogenase	655.60	H
*sucA*	α‐oxoglutarate dehydrogenase E1 component	694.23	H
*sucB*	α‐oxoglutarate dehydrogenase E2 component	416.95	M
*sucD*	Succinyl‐CoA synthetase alpha subunit	665.10	H
*sucC*	Succinyl‐CoA synthetase beta subunit	138.34	M
*frd*‐*1*	Succinate dehydrogenase	40.65	L
*frd*‐*2*	Succinate dehydrogenase	721.62	H
*frd*‐*3*	Succinate dehydrogenase	1,212.02	H
*fumC*	Fumarate hydratase	213.36	M
*mdH*	Malate dehydrogenase	1,383.80	H
*accB*	Acetyl‐CoA carboxylase biotin carboxyl carrier protein	11.19	L
*accC*	Acetyl‐CoA carboxylase, biotin carboxylase subunit	32.80	L
*accA*	Acetyl‐CoA carboxylase carboxyl transferase subunit alpha	93.49	L
*accD*	Acetyl‐CoA carboxylase carboxyl transferase subunit alpha	176.19	M
*fabD*	Acyl‐carrier‐protein S‐malonyltransferase	88.63	L
*acpP*	Acyl carrier protein	473.00	M
*acpP*‐*1*	Acyl carrier protein	134.37	M
*fabF*	3‐oxoacyl‐acyl‐carrier‐protein synthase II	113.46	M
*fabH*	3‐oxoacyl‐acyl‐carrier‐protein synthase III	162.73	M
*fabH*‐*1*	3‐oxoacyl‐acyl‐carrier‐protein synthase III	83.42	L
*fabG*	3‐oxoacyl‐acyl‐carrier protein reductase	165.28	M
*fabG*‐*1*	3‐oxoacyl‐acyl‐carrier protein reductase	60.93	L
*fabG*‐*2*	3‐oxoacyl‐acyl‐carrier protein reductase	67.52	L
*fabG*‐*3*	3‐oxoacyl‐acyl‐carrier protein reductase	142.31	M
*fabG*‐*4*	3‐oxoacyl‐acyl‐carrier protein reductase	46.28	L
*fabG*‐*5*	3‐oxoacyl‐acyl‐carrier protein reductase	168.87	M
*fabZ*	3‐hydroxyacyl‐acyl‐carrier‐protein dehydratase	188.72	M
*fabZ*‐*1*	3‐hydroxyacyl‐acyl‐carrier‐protein dehydratase	65.54	L
*fabI*	Enoyl‐acyl‐carrier protein reductase I	151.65	M
*fabL*	Enoyl‐acyl‐carrier protein reductase III	100.02	M
*ilvB*	Acetolactate synthase large subunit	480.96	M
*ilvH*	Acetolactate synthase small subunit	121.19	M
*ilvB*‐*1*	Acetolactate synthase I/II/III large subunit	1844.90	H
*ilvC*	Ketol‐acid reductoisomerase	732.24	H
*ilvD*	Dihydroxy‐acid dehydratase	149.41	M
*leuA*	2‐isopropylmalate synthase	729.44	H
*leuB*	3‐isopropylmalate dehydrogenase	695.53	H
*leuC*	(R)‐2‐methylmalate dehydratase large subunit	1,120.36	H
*leuD*	(R)‐2‐methylmalate dehydratase small subunit	129.54	M
*AspB*	Aspartate aminotransferase	1910.50	H
*yhdR*	Aspartate aminotransferase	874.50	H
*ilvE*	Branched‐chain amino acid aminotransferase	263.97	M
*ilvE‐1*	Branched‐chain amino acid aminotransferase	215.51	M
*pdhD*	Dihydrolipoamide dehydrogenase	74.71	L
*bkdA1*	Pyruvate dehydrogenase E1 component alpha subunit	74.46	L
*bkdA2*	Pyruvate dehydrogenase E1 component beta subunit	63.71	L
*bkdB*	Pyruvate dehydrogenase E2 component	132.29	M
*srfAD*	External thioesterase TEII	3,396.47	H
*srfAC*	Surfactin family lipopeptide synthetase C	3,883.08	H
*srfAB*	Surfactin family lipopeptide synthetase B	3,831.80	H
*srfAA*	Surfactin family lipopeptide synthetase A	3,090.12	H
*sfp*	4′‐phosphopantetheinyl transferase	171.40	M

Note

H: high expression (RPKM ≥ 500); L: low expression (RPKM < 100); M: medium expression (100 ≤ RPKM < 500).

### 3.5 Construction of the KEGG pathway map for surfactin biosynthesis

**Figure 6:**
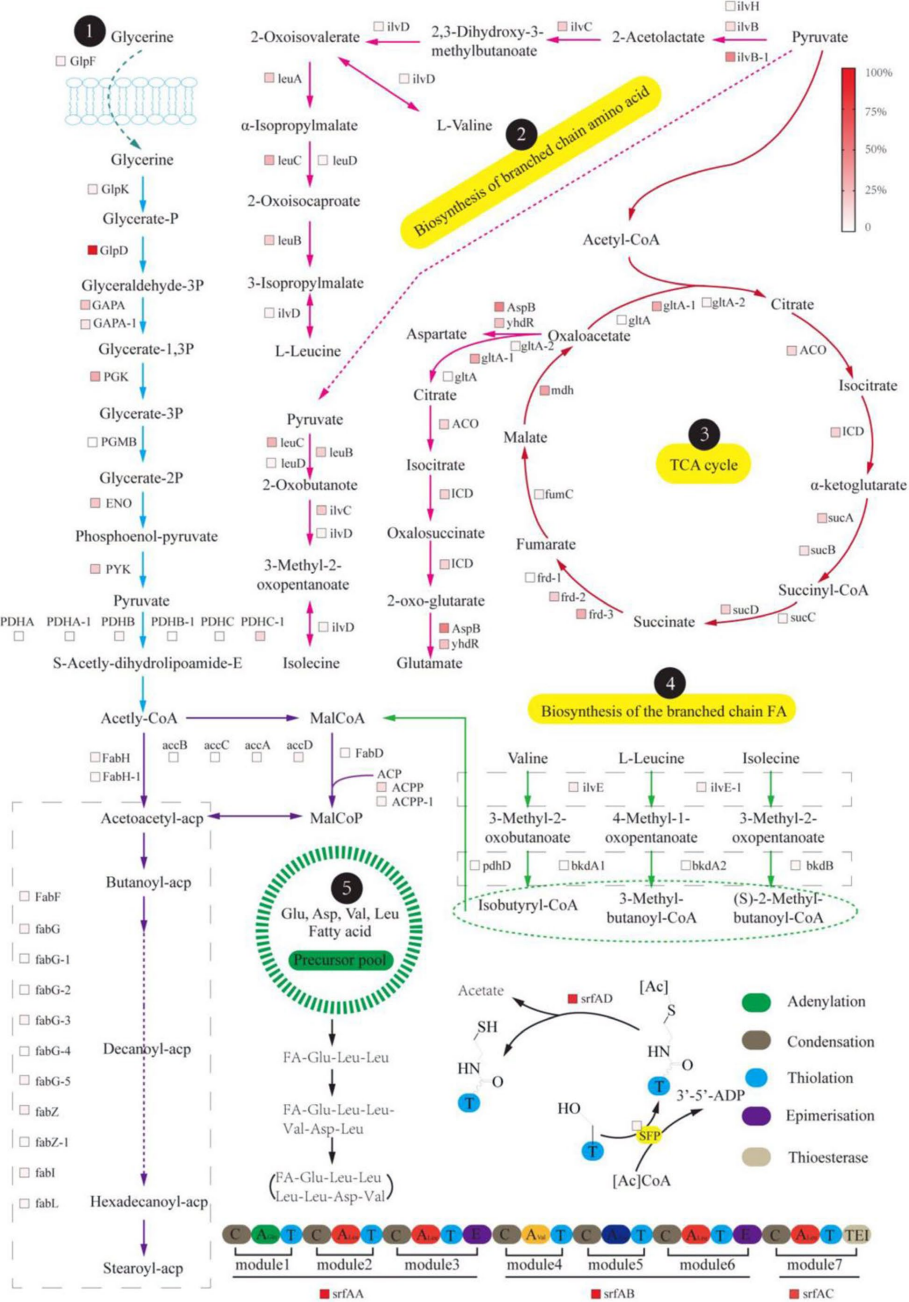
Metabolic map of the pathways responsible for surfactin biosynthesis. The metabolic network was constructed based on KEGG pathway analysis. Five modules were partitioned according to their functions. 1, 2, 3, 4, and 5, respectively, represent different metabolic modules: 1, glycolysis metabolism module; 2, branched‐chain amino acid metabolism module; 3, tricarboxylic acid cycle module; 4, branched‐chain fatty acid biosynthesis module; and 5, modular enzymatic synthesis of surfactin module. Transcriptional levels of the relevant genes in BS‐37 are show next to the pathway as a heat map, based on the ranking of expression intensity (RPKM) in the whole genome. The color of each square represents the strength of gene transcription. The transcription level (RPKM) of glpD is defined as 100%, corresponding to pure red. The expression intensity of other genes was calculated as the ratio of their respective transcription levels (RPKM) to that of glpD. The culture condition is MMG medium without d/l‐leu. The expression is the ratio of the gene to glpD. In the paper, these have been defined

In order to better understand the biosynthesis of surfactin in glycerol medium, we reconstructed the whole metabolic pathway of surfactin synthesis using the transcriptome of BS‐37 grown on glycerol. The transcripts with different levels were summarized into a heat map and subsequently displayed as a metabolic pathway map (Figure 6). The depth of the color in the map represents the strength of gene transcription according to the ranking of all the gene transcriptional intensities in the cells, represented by RPKM (Table 6). The transcripts were assessed according to their transcription levels and classified into high expression (RPKM ≥ 500), medium expression (100 ≤ RPKM < 500), and low expression (RPKM < 100) groups. According to the function, all modules were divided into glycolysis metabolism module, tricarboxylic acid cycle module, branched‐chain amino acid metabolism module, branched fatty acid biosynthesis module, and modular enzymatic synthesis of surfactin module.

Modules 1, 2, and 3 of the pathway correspond to glycerol utilization, the subsequent glycolysis pathway, and TCA cycle, which provide the basis for the metabolism of the cell. The genes involved in the utilization of glycerol, including *glpF*, *glpK,* and *glpD*, were relatively highly expressed (Holmberg, Beijer, Rutberg, & Rutberg, 1990). Particularly, the transcription level (RPKM) of *glpD*, encoding glycerol‐3‐phosphate dehydrogenase, which is the key enzyme in the glycolysis pathway when cells are grown on glycerol, reached 3,913.68, which puts it into the top 1% of all transcription levels. Most of the genes involved in glycolysis and the TCA cycle were also highly expressed to provide enough energy and carbon moieties for downstream metabolic pathways. These include pyruvate, oxaloacetate, and acetyl‐CoA, which can act as precursors participating in the biosynthesis of Val/Leu, Glu/Asp, and fatty acids, respectively.

The metabolism of branched‐chain amino acids has a significant effect on the synthesis of surfactin, since three different branched‐chain amino acid components are found in the cyclopeptide ring of surfactin. Especially, the content of Leu in the cells determines the yield of surfactin, because the peptide moiety of surfactin contains four leucines. Module 2 encompasses the synthesis of amino acids in the peptide moiety, including Glu, Val, Ile, Leu, and Asp. Genes involved in the synthesis of Leu (*leuA*, *leuB*, *leuC*) were highly expressed, supplying abundant precursors for surfactin overproduction.

The metabolism of intracellular fatty acids is divided into straight‐chain fatty acid synthesis and branched‐chain fatty acid synthesis. The precursor of straight‐chain fatty acids is acetyl‐CoA, while the precursors of branched‐chain fatty acids are catabolic products of l‐Val, l‐Leu, and l‐Ile (Kaneda, 1991). These are converted into three different branched‐chain CoAs by the action of aminotransferase (ilvE) and the branched‐chain amino acid dehydrogenase complex (BkdA, BkdB, PdhD). The branched‐chain CoAs (isobutyryl‐CoA, 3‐methylbutanoyl‐CoA and (S)‐2‐methylbutanoyl‐CoA) act as precursors for the biosynthesis of different branched‐chain fatty acids, which are another intrinsic component of surfactin, constituting the hydrophobic tail. The expression level of genes related to the degradation of branched‐chain amino acids and synthesis of branched‐chain fatty acids from module 4 was medium or low, implying a likely bottleneck of surfactin production in BS‐37.

The biosynthesis of surfactin is mainly catalyzed by NRPS, which is composed of adenylation, condensation, and thiolation domains. The biosynthesis is started with the condensation of fatty acids and Glu (And & Marahiel, 2005). Both l‐ and d‐Leu are found in the peptide chain of surfactin. The SrfAA and SrfAB proteins have the “E” function, meaning the epimerization of l‐ to d‐Leu. We supposed that the adenylation (A) domains present in SrfAA, SrfAB, and SrfAC for the activation of Leu are highly specific for l‐Leu, while d‐Leu, a structural analogue of l‐Leu, can influence the surfactin synthesis activity through competitive inhibition of the adenylation (A) domains of the Leu module. This may explain why l‐Leu supplementation can improve the production of surfactin by providing substrate molecules, while d‐Leu sharply decreased it through competitive inhibition. Notably, the competitive inhibition effects of d‐Leu could be relieved by l‐Leu supplementation (Figure 3d).

Furthermore, many studies on replacing the promoter of the *srfA*operon to abrogate the effect of quorum sensing on the synthesis of surfactin have been reported (Jiao et al., 2017; Sun et al., 2009; Willenbacher et al., 2016). Unfortunately, the results of these attempts were far from satisfactory and did not lead to high surfactin yields, indicating that a complex regulatory network controls *srfA* expression, so that modifying the *srfA* operon is not a good choice (Jung et al., 2012). In module 5, the *srfA* operon (*srfAA*, *srfAB*, *srfAC*, *srfAD*) was expressed at a very high level in BS‐37, implying that the native surfactin synthase (SrfA) promoter of BS‐37 is a strong promoter (Guan et al., 2015).

## 4 CONCLUSIONS

Transcriptional profiling of *B. velezensis* BS‐37 grown in glycerol medium showed that the expression levels (RPKM) of genes related to surfactin synthesis, encompassing the modular surfactin synthase, glycerol utilization pathway, and branched‐chain amino acid (BCAA) synthesis pathway, were all at relatively high levels. Furthermore, adding d‐/l‐Leu to the cultures did not affect the expression of genes associated with these pathways and the competitive inhibition effects of d‐Leu could be relieved by l‐Leu supplementation, implying that l‐Leu improved surfactin production by acting solely as a precursor or substrate, while d‐Leu decreased the yield by acting as a competitive inhibitor. The high transcription levels of genes encoding modular surfactin synthase, the glycerol utilization pathway, and branched‐chain amino acid (BCAA) synthesis pathways can provide great support for the establishment of a *B. velezensis* cell factory for the production of surfactin with high yield and at low cost.

## CONFLICT OF INTEREST

None declared.

## AUTHORS CONTRIBUTION

Dayuan Zhou performed the experiments and edited the primary version of the manuscript. Fangxiang Hu performed the experiments and edited the final version of the manuscript. Shuang Li was the project manager and internal reviewer. Dayuan Zhou and Fangxiang Hu contributed equally.

## ETHICS STATEMENT

None required.

## Data Availability

Genome data are deposited into GenBank (accession number:CP023414.1). The transcriptome and expression data have been deposited to Figshare (https://figshare.com/s/9955b905bb5347c23b09).
